# Female Reproductive Health Disturbance Experienced During the COVID-19 Pandemic Correlates With Mental Health Disturbance and Sleep Quality

**DOI:** 10.3389/fendo.2022.838886

**Published:** 2022-04-01

**Authors:** Michelle Maher, Aedín O’ Keeffe, Niamh Phelan, Lucy Ann Behan, Sonya Collier, David Hevey, Lisa Owens

**Affiliations:** ^1^ Department of Endocrinology, St. James’s Hospital, Dublin, Ireland; ^2^ School of Medicine, Trinity College Dublin, Dublin, Ireland; ^3^ Department of Endocrinology, Tallaght University Hospital, Dublin, Ireland; ^4^ Psychological Medicine Service, St. James’s Hospital, Dublin, Ireland; ^5^ School of Psychology, Trinity College Dublin, Dublin, Ireland

**Keywords:** COVID-19, reproductive health, menstrual abnormalities, libido, dysmenorrhea, heavy menstrual bleeding, oligomenorrhea/amenorrhea, psychological distress

## Abstract

The COVID-19 pandemic has adversely affected population mental health. Periods of psychological distress can induce menstrual dysfunction. We previously demonstrated a significant disruption in women’s reproductive health during the first 6 months of the pandemic. The present study investigates longer-term reproductive and mental health disturbances. A cross-sectional online survey was completed by 1335 women of reproductive age in April 2021. It included validated standardized measures of depression (PHQ-9), anxiety (GAD-7) and sleep quality (PSQI). 581 (56%) of women reported an overall change in their menstrual cycle since the beginning of the pandemic. There was no change in median cycle length [28 days (28-30)] or days of menses [5 (4-5)], but there was a wider variability in minimum (p<0.0001) and maximum (p<0.0001) cycle length. There was a significant increase in heavy menstrual bleeding, painful periods and missed periods compared to pre-pandemic (all p<0.0001). 64% of women reported worsening pre-menstrual symptoms. Rates of severe depression, anxiety and poor sleep were more than double those from large scale representative community samples. Poor sleep quality was an independent predictor of overall change in menstrual cycle (OR=1.11, 95%CI 1.05-1.18), and missed periods (OR=1.11, 95%CI 1.03-1.19) during the pandemic. Increased anxiety was independently associated with a change from non-painful to painful periods (OR=1.06, 95%CI 1.01-1.11) and worsening of pre-menstrual symptoms (OR=1.06, 95%CI 1.01-1.07) during the pandemic. The COVID-19 pandemic continues to bear a significant impact on female reproductive health. Increased levels of psychological distress and poor sleep are associated with menstrual cycle disruption.

## Introduction

The effect of the COVID-19 pandemic itself as well as associated public health restrictions have had an adverse effect on population mental health (1, 2) likely attributable to a combination of infection concerns, uncertain prognoses, inadequate information, economic uncertainty, and social isolation. A cross-sectional study of 847 members of the Irish public between March and June 2020, during mandatory restrictions, demonstrated significant increases in stress, depression and anxiety compared to before restrictions (1). A longitudinal cohort study performed in the UK discovered that by late April 2020, mental health had deteriorated relative to pre-pandemic trends (2). Indeed, the COVID-19 pandemic may have exacerbated gender-linked mental health challenges, and women who are pregnant, postpartum, or miscarrying are at particularly high risk for disturbance of their mental health (3).

The longer-term impact of the pandemic on mental health remains to be seen. However, pandemic-related psychological distress may persist for months following its resolution (4), therefore mental health disturbance may persist long after the current pandemic.

It is well known that periods of stress and psychological distress can induce menstrual dysfunction. Psychosocial stress triggers activation of the hypothalamic-pituitary-adrenal (HPA) axis resulting in inhibition of the hypothalamic-pituitary-gonadal (HPG) axis, with diminished gonadotrophin-releasing hormone (GnRH) pulsatility (5). This can lead to functional hypothalamic amenorrhoea (FHA), chronic anovulation without an identifiable structural cause (6). FHA is also a recognised complication of disordered eating, low body weight, and excessive exercise (7, 8). Psychological disturbance is also associated with increased menstrual-related symptoms including dysmenorrhoea (9, 10) pre-menstrual symptoms (PMS) (11), and heavy menstrual bleeding (12). Furthermore, higher stress levels are predictive of diminished libido in women (13).

We conducted a previous large-scale observational study of women of reproductive age, in September 2020, 6 months into the pandemic, which demonstrated a significant disruption in female reproductive health since the beginning of the COVID-19 pandemic (14). These disturbances were associated with a significant increase in suffering from mental health symptoms. Women who experienced low mood, anxiety and/or significant stress were more likely to report an overall change in their menstrual cycle, as well as exacerbation of pre-menstrual symptoms, more painful periods, and decreased libido. We wished to investigate the longer term impact of the pandemic on reproductive health, and how this relates to mental health, as determined by validated psychological assessment tools. Therefore, in April 2021, approximately 1 year into the pandemic, we performed a further observational study, to assess menstrual cycle, libido, lifestyle changes, and mental health symptoms in the female population.

## Materials and Methods

### Study Design

This cross-sectional online survey conforms to the ‘Checklist for Reporting Results of Internet E-Surveys (15) and is reported according to the ‘Strengthening the Reporting of Observational Studies in Epidemiology’ guidelines (16). A digital survey was created using the Google Forms platform (https://www.google.com/forms). A web link to the survey was shared by the authors *via* social media (Twitter, Instagram), both to their immediate followers, and to a wider audience through resharing of the survey. All women of reproductive age living in any country affected by the COVID-19 pandemic were invited to participate voluntarily. No incentives were offered to partake in the survey.

The survey was distributed over a 2 week period in April 2021. A 2 week interval was selected given the rapidly changing nature of the pandemic and its associated restrictions at this time, along with the high numbers of survey responses. It contained 55 questions on demographic information, reproductive health, lifestyle patterns, and mental health symptoms before and during the course of the pandemic. It also contained 4 validated questionnaires pertaining to mental health: Patient Health Questionnaire-9 (PHQ-9), General Anxiety Disorder-7 (GAD-7), sleep quality; Pittsburgh Sleep Quality Index (PSQI), and health-related quality of life; 12-item Short Form-12 Health Survey (SF-12) version 1.0.

The PHQ-9 is a 9-item screening tool that asks participants to select 1 of 4 responses about the frequency of depressive symptoms during the previous 2 weeks and consists of the criteria upon which the diagnosis of DSM-IV depressive disorders is based (17). Its score can range from 0 to 27, with those scoring ≥ 10 classified as having moderate, moderately severe, or severe depressive symptoms (17). The PHQ-9 has been well validated as a diagnostic tool for mental disorders in previous large-scale studies (18, 19). The GAD-7 contains 7 items related to the frequency of anxiety symptoms in the preceding 2 weeks, producing a total score between 0 to 21 (20). Cut-off scores of 5, 10 and 15 represent mild, moderate and severe levels of anxiety, respectively (20). It is a valid tool for the screening of generalised anxiety disorder, and the assessment of its severity (20, 21). The PSQI is a well-established instrument for the assessment of sleep quality, and consists of 19 individual items which generate 7 component scores including subjective sleep quality, sleep latency, sleep duration, sleep efficiency, sleep disturbances, use of sleep medications, and daytime dysfunction (22, 23). The sum of the scores for these 7 dimensions yields a global PSQI score, ranging from 0 to 21, and a score of ≥ 5 indicates poor sleep quality (22). The SF-12 (version 1.0) is a health-related quality of life (HRQoL) questionnaire, which generates 2 summary scores, both a mental component score (MCS-12) and a physical component score (PCS-12) (24). A score of 50 for each component score represents the United States population mean at the time of original publication in 1994 (24). The standard deviation is 10 points, with scores above 50 reflecting a HRQoL superior to that of the United states norm, and scores below 50 reflecting a poorer HRQoL relative to the norm.

The survey was distributed over 10 pages, and the numbers of questions per page varied between 3 to 27. The majority of questions were mandatory, meaning the participant could not progress to the next page unless the question was answered. Respondents were not able to review nor their change their answers on survey completion. The survey took approximately 15 to 20 minutes to complete.

### Ethical Considerations

Participants were provided with the option to remain anonymous or to volunteer their name and contact details with their consent to be contacted for involvement in future studies. They were informed as to the nature of the investigators and the purpose of the study. Ethical approval was granted by the St James’s and Tallaght University Hospital Research Ethics Committee (JREC 2020-10-CA-10). A full list of survey questions can be shared on request to the corresponding author.

### Study Participants

In total, 1335 women completed the survey. Duplicate database entries having the same user identification were eliminated before analysis, in which case the first entry was kept for analysis. BMI data and menstrual cycle data were excluded from women who conceived or delivered a baby at any point during the pandemic (n = 187). Menstrual cycle data was also excluded from women who reported that they were amenorrhoeic for any cause (e.g. secondary to pregnancy, intrauterine system, intrauterine device, implant) (n = 113).

### Statistical Analysis

Data was analysed using GraphPad Prism version 8.4.3 and SPSS version 26. Data were screened for normality using histograms, box plots, skewness and kurtosis values. Where appropriate, normally distributed continuous data are summarised using means and standard deviations (SD), and non-normal data are summarised using medians and interquartile ranges (IQR). One sample t-tests were used to compare mean values on continuous scales with published norms. Wilcoxon matched pairs rank test was used to compare non-parametric paired data. Bivariate relationships between categorical variables were assessed using c^2^ tests. Logistic regression modelling was used for categorical outcomes and multiple regression was used for continuous outcomes. For all analyses, statistical significance was set at *p* < 0.05.

## Results

### Patient Demographics 

A total of 1335 women completed the survey. The median age of the women was 34 (IQR 29-38). The participants demographic information is summarised in (**Table 1**). The median self-reported body mass index (BMI) was 24.6kg/m^2^ (22-28). 1,316/99% of the participants were of white ethnicity and 1,273/95% were living in Ireland. 652/47% were married, 298/22% were cohabiting, 369/28% were single, and 15/1% were separated or divorced. 578/43% reported that they had children, and 65/4.9% were currently breastfeeding. 1,132/85% of these women were working during the pandemic, 727/54% in the workplace and 405/30% working from home. Of those women who had children, 317/55% were home-schooling them when schools were closed and 340/59% were providing childcare while also working. 439/33% of participants were healthcare workers (HCWs), including 103/8% doctors, 132/10% nurses, and 204/16% other HCWs. 217/16% reported a significant medical history including 96/7% with Polycystic Ovarian Syndrome (PCOS), 24/2% with premature ovarian insufficiency (POI), 7/0.5% with hypothalamic amenorrhoea, and 90/7% with a thyroid disorder. 111/8% reported that they had contracted COVID-19 and tested positive, whereas 63/5% had symptoms of COVID-19 but did not get tested or did not test positive.

**Table 1:** Participant demographics/medical history.

Parameter	n/% (n=1335)
Median age (years)(IQR)	34 (29-38)
Median BMI (kg/m^2^)(IQR)	24.6 (22-28)
Ethnicity	White Irish 1,246/93% White non Irish 70/5% Asian 14/1% Black 0/0% Other 5/0.3%
Location	Ireland 1273/95% UK 55/4% Other countries 7/0.5%
Marital status	Married 652/47% Single 369/28% Cohabiting 298/22% Separated/Divorced 15/1% Widowed 1/0.07%
Occupation	Healthcare workers (HCWs) 439/33% Doctors 103/8% Nurses 132/10% Other HCWs 204/16%
Work status and location during pandemic	Full time in the workplace 606/45% Full time from home 360/27% Part time in the workplace 121/9% Part time from home 45/3% Maternity leave 83/6% Unemployed before pandemic 63/5% Furloughed 23/2% Made redundant/unemployed during pandemic 34/3%
Participants with children	578/43%
Currently breastfeeding	65/4.9%
Home schooling when schools closed	317/24%
Pre-existing medical conditions	Polycystic Ovarian Syndrome 96/7% Excess unwanted hair 427/32% Endometriosis 66/5% Premature ovarian insufficiency 24/2% Hypothalamic amenorrhoea 7/0.5% Osteopaenia/Osteoporosis 14/10% Acne 195/15% Thyroid disorder 90/7%
COVID-19 history ‘Did you have COVID-19?’	Yes, and tested positive 111/8% Yes, had symptoms but did not get tested or did not test positive 63/5% No 960/72% No, but I had contact with a confirmed case 201/15%

### Menstrual History

1095/82% of women stated that they had regular periods prior to the COVID-19 pandemic. Menstrual history is described in **Table 2**. 966/77% of those who had periods recorded them using an app, diary, smartphone, or other recording method menstrual history is described in (**Table 2**). 929/69% of participants were not using hormonal contraception or an intrauterine device. The median usual (pre-pandemic) cycle length was 28 days (28-30), with a 5 day bleed (4-5). The minimum usual cycle length was 27 days (25-28), and the maximum usual cycle length was 30 days (28-32). 128/12% of women stated that they missed periods, 100/9.7% reported missing them occasionally and 49/5% reported missing them often. 412/40% reported heavy periods and 430/42% reported painful periods.

**Table 2:** Participant self-reported menstrual cycle patterns and contraceptive use.

Parameter	n/% (n=1335)
Hormonal contraceptive/copper intrauterine device use	None 923/69% Combined contraceptive pill 211/16% Hormonal intrauterine system 91/7% Progesterone only pill 53/4% Copper intrauterine device 24/2% Implant 29/2% Depot 4/0.2%
Cycles recorded using app/diary/smartphone/other	Yes 966/77% No 286/23%
Regular periods under normal circumstances	Yes 1095/82% No 143/11% Not applicable 97/7%
Median cycle length (days)(IQR) (n=1035)	28 (28-30)
Median no. of days of menses (IQR)	5 (4-5)
Minimum length of cycle (days) (median, IQR)	27.5 (25-28)
Maximum length of cycle (days) (median, IQR)	30 (28-32)
Missed periods	128/12% (occasionally 100/9.7% often 28/2.7%)
Heavy periods	412/40%
Painful periods	430/42%

### Menstrual Cycle Changes During the COVID-19 Pandemic 

581/56% of women who had periods reported an overall change in their menstrual cycle during the COVID-19 pandemic. Summary of menstrual cycle changes is reported in **Table 3**. The duration of change in the menstrual cycle was less than 6 months for 226/32% women, between 6 months and a year for 311/44% of women, and greater than a year (i.e. for the duration of the pandemic) for 175/25% women. The median cycle length was 28 days (26-30), which was unchanged compared to before the pandemic. The median number of days of menses was 5 days (4-6), which was similar to before the pandemic (5 days, 4-5) but with a significantly wider range (p<0.0001). The minimum length of the cycle was 27.5 days (25-28), which was similar to before the pandemic, but with a significantly wider range of cycle duration (23-28, p<0.0001). Likewise, the maximum cycle length was 30 days (28-32), similar to pre-pandemic but the range was significantly wider (28-35, p<0.0001).

**Table 3:** Self-reported menstrual cycle patterns during the COVID-19 pandemic compared to recall of menstrual cycle patterns before the COVID-19 pandemic.

Parameter	Entire cohort (excluding amenorrhoeic women) n/% (n=1035)	p-value*	Women not using hormonal contraception n/% (n=722)	p-value*
Overall change in menstrual cycle	Yes 581/56% No 454/44%		Yes 411/59% No 288/41%	
Duration of change in menstrual cycle	< 6 months 226/32% 6-12 months 311/44% > 1 year 175/25%		< 6 months 155/31% 6-12 months 216/44% > 1 year 124/25%	
Change in libido/sex drive (n=1335)	No change 456/34% Increased libido-163/12% Decreased libido-716/54%		No change 246/34% Increased libido-92/12% Decreased libido-384/53%	
Change in premenstrual symptoms (PMS)	PMS unchanged 281/28% PMS better 52/5% PMS worse 658/66%		PMS unchanged 194/29% PMS better 41/6% PMS worse 444/65%	
Median cycle length (days) (IQR)	28 (26-30)	0.36	28 (26-31)	0.78
Median no. of days of menses (IQR)	5 (4-6)	<0.0001	5 (4-6)	0.0014
Minimum length of cycle (days) (median, IQR)	27 (23-28)	<0.0001	26 (24-28)	0.001
Maximum length of cycle (days) (median, IQR)	30 (28-35)	<0.0001	31 (28-35)	<0.0001
Missed periods	210/20% (+82/8%) (occasionally 128/12%, often 82/8%) Median no. missed = 2(2-4)	<0.0001	141/20% (+63/9%) (occasionally 87/12%, often 54/8%) Median no. missed = 2 (2-4)	<0.0001
Heavy periods	522/50%	<0.0001	385/55%	<0.0001
Painful periods	605/59%	<0.0001	433/62%	<0.0001

*Analysis compares self-reported data before and during the pandemic for each parameter.

163/12% of women surveyed reported an increase in their libido, whereas 716/54% reported a decrease in their libido. 658/66% reported a worsening of their premenstrual symptoms (PMS), whereas 52/5% believed that their PMS had improved. 210/20% missed periods during the pandemic, 128/12% reported missing them occasionally and 82/8% reported missing them often. Overall, this was 82/8% more than prior to the pandemic (p<0.0001). The median number of missed periods was 2 (2-4). 522/50% reported heavy periods, 110/10% more than before the pandemic (p<0.0001). 605/59% reported painful periods, 175/17% more than before the pandemic (p<0.0001).

Subgroup analysis showed that women not using hormonal contraception were even more likely to report an overall change in their menstrual cycle (411/59%). 141/20% of this group missed periods during the pandemic, 87/12% reported missing them occasionally and 54/8% reported missing them often. Overall, this was 63/9% more than pre-pandemic (p<0.0001), and the median number of miser periods was 2 (2-4).This group were also more likely to report heavy periods (385/55%, p<0.0001) or painful periods (433/62%, p<0.0001) during the pandemic. Unemployed women were significantly more likely to report an overall change in their menstrual cycle than women in employment (p=0.0014). However, they did not experience heavier periods (p=0.09), or an increase in missed periods (p=0.19). There was no difference in overall menstrual cycle change in those who contracted COVID-19 and those who did not (p=0.48), however a relatively small proportion of this cohort tested positive for the virus.

### Lifestyle and Mental Health Change During the COVID-19 Pandemic

The median change in self-reported weight over the course of the pandemic was an increase of 2kg (0-5, p<0.0001). 623/58% of women gained weight, and 275/25% of women lost weight **(** **Table 4** **).** Women reported carrying out a median of 164 minutes (90-280) of exercise a week at the time of being surveyed, an increase of 20 minutes (-60-100, p<0.0001) compared to before the pandemic. 713/53% thought that their diet had worsened during the pandemic (**Table 4**), whilst 324/24% felt that their diet overall had improved (**Table 4**). 614/46% of women reported working more since the beginning of the pandemic, whereas 221/17% stated they were working less (**Table 4**).

**Table 4:** Self-reported lifestyle changes during the COVID-19 pandemic compared to pre-pandemic lifestyle patterns.

Parameter	n/% (n = 1,335)	p-value
Change in weight (kg) (median, IQR)	+2 kg (0-5) No change in weight 185/17% Weight loss 275/25% Weight gain 623/58%	<0.0001
Change in minutes of exercise per week (median, IQR)	+20 minutes (-60-100)	<0.0001
Type of exercise	Walking 1,224/92% Running 338/25% Strength training 281/21% Yoga/Pilates 328/25% HiiT 300/22% Other 131/10% None 27/2%	
Diet	Overall diet is unchanged 298/22% Overall diet is better 324/24% Overall diet is worse 713/53%	
Change in work practice	No change 500/37% Working more 614/46% Working less 221/17%	

Respondents reported a significant increase in suffering from mental health symptoms compared to symptoms they reported experiencing before the pandemic **(** **Table 5** **).** 1145/86% of women reported suffering at least one new symptom during the pandemic, including low mood (687/51%, p<0.0001), anxiety (599/45%, p=0.02), poor sleep (697/52%, p<0.0001), significant stress (150/30%, p<0.0001), binge eating (412/31%, p<0.0001), poor concentration (473/35%, p<0.0001), loneliness (516/39%, p<0.0001), poor appetite (192/14%, p<0.0001), and excess alcohol use (116/9%, p<0.0001) (**Table 5**). In addition, 867/65% of women reported worsening of a pre-existing mental health symptom during the pandemic, including low mood (367/27%), anxiety (48/37%), poor sleep (372/28%), significant stress (255/19%), binge eating (217/16%), poor concentration (192/14%), loneliness (245/18%), poor appetite (68/0.5%), excess alcohol use (55/4%), and illicit drug use (4/0.3%) (**Table 5**).

**Table 5:** Self-reported mental health symptoms comparing pre-COVID-19 pandemic to April 2021, during the COVID-19 pandemic.

N=1335	Pre-existing mental health symptoms (n/%)	New mental health symptom during pandemic (n/%)	Worsening of pre-existing mental health symptoms during pandemic (n/%)	P values*
Any mental health symptom/symptoms	904/68%	1145/86%	867/65%	<0.0001
Low mood	429/32%	687/51%	367/27%	<0.0001
Anxiety	545/41%	599/45%	48/37%	0.02
Poor sleep	418/31%	697/52%	372/28%	<0.0001
Significant stress	300/22%	510/30%	255/19%	<0.0001
Binge eating	259/19%	412/31%	217/16%	<0.0001
Poor concentration	196/15%	473/35%	192/14%	<0.0001
Loneliness	166/12%	516/39%	245/18%	<0.0001
Poor appetite	90/7%	192/14%	68/0.5%	<0.0001
Excess alcohol use	44/3%	116/9%	55/4%	<0.0001
Illicit drug use	9/0.7%	4/0.3%	4/0.3%	0.18

^*^Analysis compares self-reported data before and during the pandemic for each parameter.

The mean depression score on the PHQ-9 was 7.53 **(** **Table 6** **),** with 408/30.6% of women experiencing at least moderate depressive symptoms, as compared to 10% of women in large scale representative community samples (25) in the United States of America. This comparator group represents 968 women, aged between 18 to 54 years surveyed from 2005 to 2008 as part of the National Health and Nutrition Examination Survey (NHANES). The NHANES provides generalizable data as it uses a complex sampling design to obtain a representative sample of the civilian, noninstitutionalized population of the United States.

**Table 6:** Mental health survey results of participants completed in April 2021.

Depression: Patient Health Questionnaire (PHQ-9): Mean = 7. 53; SD = 5.09^a^, n= 1335	
Category (Score)	n/%
None (0-4)	442/33%
Mild (5-9)	485/36%
Moderate (10-14)	272/20%
Moderate-severe (15-19)	103/8%
Severe (20-27)	33/3%
**Anxiety: General Anxiety Disorder-7 (GAD-7): Mean = 6.44; SD = 5.06** ^**b**^ **, n= 1335**	
Category (Score)	n/%
None (0-4)	551/41%
Mild (5-9)	460/35%
Moderate (10-14)	218/16%
Severe (≥ 15)	106/8%
**Pittsburgh Sleep Quality Index (PSQI) global score: Mean = 9.45; SD = 2.86** ^**c**^ **, n= 1335**	
Category (Score)	n/%
Good sleep quality (0-4)	50/4%
Poor sleep quality (≥ 5)	1,252/96%

a Population with none, mild, moderate, moderately severe, and severe depressive symptoms from PHQ-9 scores.

b Population with none, mild, moderate and severe symptoms from the GAD-7 scores.

c Population with good sleep quality and poor sleep quality as determined by the PSQI score.

The mean score on the GAD-7 was 6.44 **(** **Table 6** **),** with 324/24.2% of women experiencing at least moderate anxiety symptoms, as compared to 7% of women in a large population sample in Germany (21). This sample represents 9,721 women with an age range of 18 to 80 years surveyed as part of the LIFE-Adult-Study of the Leipzig Centre for Civilisation Diseases (LIFE) between 2011 and 2014. The LIFE study is a population-based study with a representative sample of people living in Leipzig, a city in Germany which houses 550,000 inhabitants. The study targets an age and gender-stratified random selection of inhabitants, ranging in age from 18 to 80 years from the local residents’ registration office. Given that there was a lack of a significant and systematic age trend, the study presents the normative scores (cumulative percentages) for the female population without separate analyses of age groups. Thus, it is reasonable to compare our study sample to this group, which included postmenopausal women.

The vast majority of women (1252/96.2%) experienced poor sleep quality as indicated by a global PSQI score of ≥ 5 (**Table 6** **).** The rates of poor sleep were over double that from large scale representative population samples in Germany pre-pandemic (23). Similar to the comparator group we described previously for the GAD-7 score, this sample is generated from the LIFE study conducted in Leipzig, Germany between 2011 and 2014. It describes the sleep quality of 4,864 women aged between 18 to 80 years, of whom 42% reported poor sleep quality. Furthermore, there was no linear association between age and sleep quality, rendering it reasonable to compare this group with our younger cohort of women.

Within the SF-12 survey, our population reported a higher PCS-12 (52.01 ± 8.02) mean (better physical health-related quality of life) and a lower MCS-12 (40.50 ± 10.67) mean (poorer mental health-related quality of life) when compared with the United States norm of 50 (p<0.001) (**Table 6** **)**. The SF-12 survey was created through regression methods whereby 12 items were selected and scored from the Medical Outcomes Study 36-item short-form health survey (SF-36) to reproduce the physical component summary and mental component summary scales in the general US population in the 1990s (n = 2,333) (24). There were no age nor gender-stratified norms reported within this cohort.

Women experienced a variety of stressors, as described in **Supplementary Table 1**, the most frequent of which was work-related stress (753/56%), followed by family illness or bereavement (300/22%). On the contrary, 1,181/88% of women reported at least one positive aspect in relation to the pandemic, as outlined in **Supplementary Table 1**, the most prevalent of which was a positive aspect on personal relationships.

### Relationship Between Menstrual Cycle Changes and Lifestyle and Mental Health

**Table 7** demonstrates the relationship between menstrual cycle changes and lifestyle and mental health survey scores. Sleep quality (PSQI) predicted overall perceived menstrual cycle change using logistic regression analysis, independent of other mental health disturbance (OR 1.11, 95% CI 1.048-1.178). Each unit decrease in sleep quality increased the odds of menstrual cycle change by 12%. A poorer physical health-related quality of life score (PCS-12) was associated with heavy periods both pre-pandemic (OR 0.98, 95% CI 0.9611-0.998), and during the pandemic (OR 0.97, 95% CI 0.946-0.984), but was not found to be an independent predictor for worsening of heavy periods during the pandemic (OR 0.98, 95% CI 0.962-1.006), along with PHQ-9, GAD-7, PSQI and MCS-12 scores. Lower PCS-12 scores were found to be associated with painful periods both pre-pandemic (OR 0.97, 95% CI 0.949-0.986), and during the pandemic (OR 0.97, 95% CI 0.949-0.986), but were not predictive of a change from experiencing non-painful to painful periods over the course of the pandemic. A decreased PSQI score was associated with painful periods during the pandemic (OR 1.07, 95% CI 1.005-1.130), but was not associated with a change from non-painful to painful periods. However, increased anxiety symptoms were found to be associated with a change from non-painful to painful periods (OR 1.06, 95% CI 1.007-1.109), with each unit increase in GAD-7 increasing the odds of period pain getting worse by 6%. Poorer sleep quality was an associated with missed periods during the pandemic, with each unit decrease in the PSQI score raising the odds of missing periods by 11% (OR 1.11, 95% CI 1.029-1.189). Increased anxiety symptoms (OR 1.06, 95% CI 1.010-1.074), and poorer mental health-related quality of life (OR 0.97, 95% CI 0.953-0.993) were both associated with worsening of pre-menstrual symptoms (PMS) during the pandemic. Each unit increase in the GAD-7 score increased the chances of PMS getting worse by 6%, whereas each unit increase in the MCS-12 (i.e. higher levels of mental health functioning) lowered the odds of PMS worsening by 3%. Higher levels of both physical (OR 0.98, 95% CI 0.961-0.994) and mental health-related quality of life (OR 0.98, 95% CI 0.959-0.991) were protective of worsening libido during the pandemic. Every unit increase in the PCS-12 and the MCS-12 lowered the odds of libido getting worse by change by 2%.

**Table 7:** Logistic regression analysis of mental health survey score as predictors of menstrual changes during the COVID-19 pandemic (n=1335).

Mental health survey	Odds ratio (OR)	95% CI
**Overall change in menstrual cycle-** c^2^(5) = 40.46, *p*<.001; Nagelkerke R^2^=.06		
Depression (PHQ9)	1.01	0.96 - 1.06
Anxiety (GAD-7)	1.02	0.98 - 1.07
Sleep Quality (PSQI)	**1.11**	**1.05 - 1.18**
Physical HRQoL (PCS-12)	1.00	0.978- 1.02
Mental HRQoL (MCS-12)	1.00	0.98 - 1.01
**Worsening of heavy periods-** c^2^(5) = 13.95 *p* <.05; R^2^ = .03		
Depression (PHQ9)	0.97	0.91 - 1.03
Anxiety (GAD-7)	1.05	0.99 - 1.10
Sleep Quality (PSQI)	1.06	0.99 - 1.14
Physical HRQoL (PCS-12)	0.98	0.96 – 1.01
Mental HRQoL (MCS-12)	0.99	0.97- 1.01
**New painful periods during pandemic-** c^2^(5) = 14.91 *p* <.05; R^2^ = .03		
Depression (PHQ9)	0.98	0.93 - 1.04
Anxiety (GAD-7)	**1.06**	**1.01 - 1.11**
Sleep Quality (PSQI)	1.05	0.98- 1.12
Physical HRQoL (PCS-12)	1.00	0.98 – 1.03
Mental HRQoL (MCS-12)	0.99	0.97- 1.02
**Missed periods-** c^2^(5) = 28.02 *p* <.001; R^2^ = .05		
Depression (PHQ9)	1.04	0.98 - 1.10
Anxiety (GAD-7)	1.01	0.96 - 1.06
Sleep Quality (PSQI)	**1.11**	**1.03- 1.19**
Physical HRQoL (PCS-12)	1.00	0.98 – 1.03
Mental HRQoL (MCS-12)	1.00	0.98- 1.02
**Worsening of PMS during pandemic-** c^2^(5) = 75.96, *p* <.001; R^2^ = .09		
Depression (PHQ9)	1.04	0.98 - 1.10
Anxiety (GAD-7)	**1.06**	**1.01 - 1.07**
Sleep Quality (PSQI)	1.01	0.95 - 1.07
Physical HRQoL (PCS-12)	0.98	0.98- 1.02
Mental HRQoL (MCS-12)	**0.97**	**0.95 – 0.99**
**Worsening of libido-** c^2^(5) = = 52.17, *p* <.001; R^2^ = .04		
Depression (PHQ9)	1.03	0.98 - 1.07
Anxiety (GAD-7)	1.01	0.97 - 1.05
Sleep Quality (PSQI)	1.00	0.96 - 1.05
Physical HRQoL (PCS-12)	**0.98**	**0.96- 0.99**
Mental HRQoL (MCS-12)	**0.98**	**0.96 – 0.99**

Bold values indicate that this mental health parameter meets statistical significance as an independent predictor of the relevant menstrual cycle change as the 95% CI does not cross 0.

## Discussion

This large-scale observational study has shown that the majority of the general female population in Ireland continue to experience reproductive health disturbance secondary to the COVID-19 pandemic. Menstrual cycle disruption is associated with increased levels of psychological distress and poor sleep, with many women also reporting longer working hours, weight gain and a deterioration in diet. Menstrual cycle disturbance precedes the introduction of mass vaccination against COVID-19 and the vast majority of our cohort did not test positive for COVID-19. We acknowledge however that the generalisability of our findings may be somewhat limited by selection bias.

The extent of psychological distress experienced by women since the outbreak of the pandemic is striking. Approximately half of participants reported low mood and anxiety, and approximately one third reported significant stress, binge eating, poor concentration, and loneliness. Almost one third of women met criteria for moderate depressive symptoms, whereas one quarter met criteria for moderate anxiety, rates of which are over triple that of large scale representative community samples in the United States of America (25) and Germany (21) pre-pandemic. Women also reported a substantial impairment in mental HRQoL when compared with population-based norms, reflective of the negative impact of mental distress on this cohort’s well-being. Depressive and anxiety symptoms were positively correlated with an overall change in menstrual cycle, but were not predictive of an overall change independent of other measures of mental health disturbance. Nearly all women reported poor sleep quality (23) the severity of which was an independent predictor of overall alteration in their menstrual cycle. It is known that reproductive hormones are under regulation by circadian rhythm (10), and that sleep disturbance may have wide implications for female reproduction as it has previously been associated with infertility and diminished ovarian reserve (26).

The negative relationship between psychological stress and menstrual health may be explained by a myriad of physiological mechanisms. Psychosocial stress results in the activation of the HPA axis, which exerts multiple inhibitory effects on the female reproductive system. Corticotropin-releasing hormone (CRH) and CRH-induced pro-opiomelanocortin peptides inhibit GnRH secretion, whereas glucocorticoids inhibit luteinizing hormone (LH) secretion from the pituitary, and oestrogen and progesterone release from the ovary (27, 28). Stress exerts a further deleterious effect on the HPG axis through the activation of a hypothalamic sympathetic neural pathway which results in norepinephrine release in the ovary (29).

These neuroendocrine aberrations are implicated in the pathophysiology of FHA whereby psychological distress and metabolic challenges, including dietary restriction, excessive exercise and weight loss, interact to disrupt GnRH drive (7, 8, 30, 31). This cohort of women demonstrated a significant increase in missed periods, possibly attributable to a combination of psychological distress and an increase in the volume of exercise being performed. However, over half of respondents reported weight gain along with a deterioration in their diet, which suggests that this amenorrhoea may not only be driven by psychological and metabolic stressors but also by increasing adiposity and worsening of PCOS symptoms (32, 33). Moreover, we found that poor sleep quality was found to be predictive of missed periods during the pandemic, independent of other measures of mental health disturbance. Sleep disturbance has been found to be more prevalent in those with FHA, in addition to an association between sleep disorders and high anxiety levels (34, 35). It is possible that sleep disturbance may play an independent role in the neuroendocrine pathways governing the HPG axis, *via* downregulation of GnRH secondary to elevated melatonin levels (35, 36) Amenorrhoeic athletes, who have similar alterations of the HPG axis to those observed in FHA, have been shown to present higher melatonin peak amplitudes, with a delay in peak onset (37). However, the precise pathophysiological mechanisms underpinning this association between amenorrhoea and sleep disturbance remain to be elucidated.

The chronicity of amenorrhoea is as yet unknown and is likely to depend on the duration of the psychological burden and corresponding lifestyle changes stemming from the pandemic, which may persist following its resolution (4). Chronic anovulation has unfavourable long-term health outcomes, including infertility, osteoporosis and adverse cardiovascular consequences (38).

Over half of women reported heavy menstrual bleeding and dysmenorrhoea, a significant increase compared to before the pandemic. These findings are expected in light of previous studies which show an association of both with low mood, stress and psychological distress (9, 10, 12). In our study, increased anxiety symptoms were an independent risk factor of change from non-painful to painful periods during the pandemic. Although there was a positive association demonstrated, anxiety symptoms did not meet statistical significance as an independent predictor for worsening of heavy menstrual bleeding.

Two thirds of women described worsening symptoms of PMS. Equally, psychosocial stress is an independent predictor for experiencing PMS (39). PMS, when severe can result in marked functional impairment, and may predispose to mental health disorders, including postnatal depression, perimenopausal depression, and anxiety disorders (40). It has been postulated that panic disorder in particular shares certain pathophysiological mechanisms with PMS (41, 42). Indeed, we found that increased anxiety symptoms and poorer mental HRQoL were both independent risk factors for worsening of PMS during the pandemic.

54% of women also reported a decrease in their libido over the course of the pandemic. Self-reported reduced sexual desire is associated with decrements in HRQoL, and negative emotional states, including hopelessness and poor self-esteem (43–45). In our study, superior levels of physical and mental HRQoL as measured by the SF-12 were protective of worsening libido, likely reflective of a bidirectional relationship between these variables. Indeed, a recent study demonstrated the negative impact of the pandemic on female sexual function, with decreases in frequency of sexual intercourse and female sexual function index (FSFI) scores, along with an increase in female sexual distress scale (FSDS) scores observed in conjunction with reduced quality of life (46). Parallel to this, pandemic-related restrictions may impede access to contraceptive supplies, or result in discontinuation of contraception by women of their own accord. A study conducted in a family planning service in Italy showed that 51% of non-cohabiting or single women had discontinued short acting reversible contraception, having independently decided to take a contraceptive break while social distancing (47). However, 15% of these women subsequently had an unplanned pregnancy. This highlights the need to ensure that sexual and reproductive health services remain a priority during the pandemic.

Women reported no change in the median length of their menstrual cycle. However, there was a wider variability in the minimum and maximum cycle length recorded, with a shortening of the minimum cycle length and a lengthening of the maximum cycle length observed. It is known that for women attempting to conceive, shorter and longer cycles are associated with lower fecundity and are more likely to result in spontaneous abortion (48, 49).

Women also described lifestyle changes which may have impacted their menstrual cycles. Despite an increase in the amount of exercise being undertaken, by 20 minutes per week, women gained a median weight of 2kg. This is likely a result of half of women reporting that their diet was worse and almost one third reporting new binge eating since the onset of the pandemic. In addition, 46% of women began working more during the pandemic, limiting their time available to prepare healthy meals. While weight gain is associated with oligomenorrhoea/amenorrhoea as previously indicated, it is also correlated with irregular menstrual cycles and heavy menstrual flow (32, 39).

While a significant proportion of women outlined the negative repercussions of the pandemic on their menstrual cycle, lifestyle and mental health, there was a minority who describes a positive influence. Some women reported an improvement in PMS and libido. There was an increase in average exercise per week, and a quarter of women both lost weight and reported an improvement in their diet. When they were invited to comment about the overall impact of the COVID-19 pandemic, 6% of women described an overall positive impact of the pandemic on their lives citing a slower pace of life, less social obligations and increased time with family as their rationale. Interestingly, 10% of women reported an overall positive impact of the pandemic when surveyed in an earlier stage of the pandemic, in September 2020 (14). The adverse effects may have outweighed these positive effects as the pandemic progressed.

The major strength of our study is the large number of women surveyed. Another strength of the study is the fact that 77% of participants were recording their menstrual cycle pattern using an app or a diary, therefore the menstrual cycle data is likely to limit recall bias. A recent study which examined raw data from over 18,000 women using a menstrual cycle tracking app did not show any evidence of population-level changes to ovulation and menstruation during the COVID-19 pandemic (50). Whilst this methodology reduces the potential for recall bias with regards to menstrual cycle data, they employ surface-level measurements of self-reported stress, which may not be representative of true psychological distress. The present study uses well validated standardized measures of psychological distress and sleep quality to strengthen our conclusions. Moreover, their study observed menstrual cycle patterns between March and September 2020, which may not have been a sufficiently long enough time period to observe significant stress-related physiological effects. Interestingly, there was a similarity in the trends observed with regards to reproductive and mental health disturbance in the current study conducted in April 2021 when compared to our previous study, which we carried out in September 2020 (14). Whilst the initial study showed that the pandemic resulted in significant female reproductive and mental health disturbance in the short-term, the current study adds to this data by demonstrating that these disturbances have persisted and, in fact, appear to have progressed over time. Given the evolving nature of the pandemic and its societal impact, ongoing assessment of the impact on women’s health throughout the pandemic and beyond is imperative. In the first study, 46% of women reported an overall change in their menstrual cycle whereas this increased to 56% in the follow-up current study. In addition, there is an increased burden of menstrual ill-health reported in the second study with an increasing prevalence of pre-menstrual symptoms (64% vs 53%), heavy menstrual bleeding (50% vs 47%) and dysmenorrhoea (59% vs 49%). It is possible that 1 year into the pandemic, when the current study was completed, that the toll that the pandemic has taken on women’s lives has been amplified. It is critically important to continue to assess and report these trends as this may impact women’s reproductive health and fertility outcomes in the longer-term.

The major novelty of our present study relative to our previous study and indeed other similar studies, lies in the addition of standardized psychological assessment tools to the survey to assess depression (PHQ-9), anxiety (GAD-7), sleep quality (PSQI) and quality of life (SF-12). These questionnaires are well-validated and provide an objective measure of the prevalence and severity of mental health disturbance, with existing population-based normative data for comparison. This standardized mental health data facilitates correlation between menstrual disturbance and mental health disturbance, using multivariate analyses. The previous study recorded subjective measures of mental health disturbance by self-reported symptoms, including low mood, anxiety and poor sleep.

There are several limitations to our study. First, our findings rely on a survey, which is subject to self-reporting bias. Self-reporting of menstrual cycle length has been shown to have measurement error (51). However, the majority of women in this study were recording their cycles using a written or electronic format, reducing recall bias. Furthermore, studies investigating the prevalence of pre-menstrual symptoms, show a consistency between retrospective and prospective studies (40). Self-reported weight can also be inaccurate; however, a study showed that online self-reporting provides acceptably reliable weight data for young adults, with modest under-estimation by an average of just 0.4kg (52). A further potential limitation of this study is that results may be vulnerable to sampling biases, where those who decided to complete the survey were those who were more likely to have experienced menstrual disturbance. Another limitation is that the population of women that completed the survey may not be representative of a larger population, with an over-representation of relatively privileged groups. The use of an online survey precludes the involvement of women who do not have internet access or do not have sufficient command of the English language for example. The majority of our cohort were of white ethnic background and were in employment, which may reduce the generalizability of the findings. In fact unemployed women in the cohort reported more menstrual cycle disturbance, so this cohort may under-represent the impact on a population level. The pandemic has disproportionally impacted women of black, Asian or minority ethnic (BAME) background, who are more likely to be severely affected if they contract COVID-19 (53), are more likely to experience higher levels of depressive symptoms in the context of the pandemic (54), and are more likely to be from lower socio-economic backgrounds (55). Therefore the widespread psychological distress identified in our study may indeed be more prevalent across the larger population. This study is also limited by the fact that individuals who are biologically female but do not identify as female may not have partaken in the study as it specifically invited women of reproductive age to participate. While the estimated proportion of gender-diverse individuals varies between 0.1 and 2% of the population (56), this group are likely to have been underrepresented in this study. A further limitation stems from the complex and multi-directional relationship between variables (57). Measures of mental health disturbance, including anxiety symptoms, depressive symptoms and sleep quality are inter-related and a larger-scale survey would be required to further explore these aetiological factors in the context of reproductive health disturbance.

This study clearly indicates that women continue to suffer significant reproductive and mental health disturbances, over a year following the outbreak of the COVID-19 pandemic.

Further longitudinal studies are needed to elucidate the longer-term reproductive and psychological consequences of the pandemic, which are likely to depend on the duration of the pandemic and vaccine efficacy. This study was conducted when the unveiling of the COVID-19 vaccination programme was at a relatively early stage and mass vaccination was not being offered to this age cohort in Ireland. We plan to conduct interval surveys to record changes in menstrual cycle, libido, lifestyle, sleep quality and mental health in this study population until after the pandemic has largely subsided. We advised participants to install a menstrual-tracking app on completion of the present survey, from which we hope to obtain menstrual cycle data in future studies, to provide greater objectivity and limit the potential for recall bias. Future work should also explore the aetiological factors which may contribute to greater levels of reproductive health disruption, including the impact of COVID-19 infection and the influences of socio-economic status and pre-existing reproductive health conditions. Further research should involve regular study visits to facilitate objective measurements of body mass index, sex hormones (to provide evidence for FHA), and markers of bone turnover (to assess if those who are experiencing FHA are experiencing bone loss). Collecting this data is crucial for studying women’s health at a population level and may be used to inform global public health policy. This work has informed us of the impact of the pandemic on this population of women to date and can be used to inform reproduction education for women and health service policymakers in relation to managing stress and menstrual and reproductive factors. it is imperative to establish psychological support services for women affected by menstrual ill-health as a result of the unprecedented psychological burden arising from the COVID-19 pandemic. These services could include psychological support workshops, digital-based mental health interventions and patient information resources. Furthermore, it is critical to harness this data to progress the awareness and education of women around the potential implications of psychosocial stressors in general on menstrual irregularities and reproduction, such that they feel empowered to impact their own reproductive health by accessing educational materials and support services.

## Data Availability Statement

The original contributions presented in the study are included in the article/**Supplementary Material**. Further inquiries can be directed to the corresponding author.

## Ethics Statement

The studies involving human participants were reviewed and approved by St James’s and Tallaght University Hospital Research Ethics Committee (JREC 2020-10-CA-10). Written informed consent for participation was not required for this study in accordance with the national legislation and the institutional requirements.

## Author Contributions

MM is the primary investigator, led the collection and assembly of the data and drafted the first version of the manuscript. AO’K assisted in the assembly of the data. NP and LA-B contributed to the design of the study. SC contributed to the selection of psychological measures employed in the survey. DH contributed to the selection of psychological measures, and carried out data analysis and interpretation. LO designed the study, completed data analysis and supervised the project. All authors revised the manuscript and approved the final submitted version.

## Conflict of Interest

The authors declare that the research was conducted in the absence of any commercial or financial relationships that could be construed as a potential conflict of interest.

## Publisher’s Note

All claims expressed in this article are solely those of the authors and do not necessarily represent those of their affiliated organizations, or those of the publisher, the editors and the reviewers. Any product that may be evaluated in this article, or claim that may be made by its manufacturer, is not guaranteed or endorsed by the publisher.

## References

[1] Burke T Berry A Taylor LK Stafford O Murphy E Shevlin M. Increased Psychological Distress During COVID-19 and Quarantine in Ireland: A National Survey. J Clin Med (2020) 9(11):3481. doi: 10.3390/jcm9113481 PMC7693396 33126707

[2] Pierce M Hope H Ford T Hatch S Hotopf M John A. Mental Health Before and During the COVID-19 Pandemic: A Longitudinal Probability Sample Survey of the UK Population. Lancet Psychiatry (2020) 7(10):883–92. doi: 10.1016/s2215-0366(20)30308-4 PMC7373389 32707037

[3] Almeida M Shrestha AD Stojanac D Miller LJ. The Impact of the COVID-19 Pandemic on Women’s Mental Health. Arch Womens Ment Health (2020) 23(6):741–8. doi: 10.1007/s00737-020-01092-2 PMC7707813 33263142

[4] Peng EY-C Lee M-B Tsai S-T Yang C-C Morisky DE Tsai L-T. Population-Based Post-Crisis Psychological Distress: An Example From the SARS Outbreak in Taiwan. J Formosan Med Assoc (2010) 109(7):524–32. doi: 10.1016/S0929-6646(10)60087-3 PMC3106105 20654792

[5] McCosh RB Breen KM Kauffman AS. Neural and Endocrine Mechanisms Underlying Stress-Induced Suppression of Pulsatile LH Secretion. Mol Cell Endocrinol (2019) 498:110579. doi: 10.1016/j.mce.2019.110579 31521706 PMC6874223

[6] Berga SL Girton LG. The Psychoneuroendocrinology of Functional Hypothalamic Amenorrhea. Psychiatr Clin (1989) 12(1):105–16. doi: 10.1016/S0193-953X(18)30454-4 2565569

[7] Laughlin GA Dominguez C Yen S. Nutritional and Endocrine-Metabolic Aberrations in Women With Functional Hypothalamic Amenorrhea. J Clin Endocrinol Metab (1998) 83(1):25–32. doi: 10.1210/jcem.83.1.4502 9435412

[8] Benson JE Engelbert-Fenton KA Eisenman PA. Nutritional Aspects of Amenorrhea in the Female Athlete Triad. Int J Sport Nutr Exercise Metab (1996) 6(2):134–45. doi: 10.1123/ijsn.6.2.134 8744786

[9] Abu Helwa HA Mitaeb AA Al-Hamshri S Sweileh WM. Prevalence of Dysmenorrhea and Predictors of its Pain Intensity Among Palestinian Female University Students. BMC Womens Health (2018) 18(1):18. doi: 10.1186/s12905-018-0516-1 29334974 PMC5769430

[10] Ibrahim NK AlGhamdi MS Al-Shaibani AN AlAmri FA Alharbi HA Al-Jadani AK. Dysmenorrhea Among Female Medical Students in King Abdulaziz University: Prevalence, Predictors and Outcome. Pak J Med Sci (2015) 31(6):1312–7. doi: 10.12669/pjms.316.8752 PMC4744273 26870088

[11] Yamamoto K Okazaki A Sakamoto Y Funatsu M. The Relationship Between Premenstrual Symptoms, Menstrual Pain, Irregular Menstrual Cycles, and Psychosocial Stress Among Japanese College Students. J Physiol Anthropol (2009) 28(3):129–36. doi: 10.2114/jpa2.28.129 19483374

[12] Morales-Carmona F Pimentel-Nieto D Bustos-López H. Menstrual Cycle Perception and Psychological Distress in a Sample of Mexican Women. Rev Invest Clin (2008) 60(6):478–85. 19378834

[13] Raisanen JC Chadwick SB Michalak N van Anders SM. Average Associations Between Sexual Desire, Testosterone, and Stress in Women and Men Over Time. Arch Sex Behav (2018) 47(6):1613–31. doi: 10.1007/s10508-018-1231-6 29845444

[14] Phelan N Behan LA Owens L. The Impact of the COVID-19 Pandemic on Women’s Reproductive Health. Front Endocrinol (Lausanne) (2021) 12:642755. doi: 10.3389/fendo.2021.642755 33841334 PMC8030584

[15] Eysenbach G. Improving the Quality of Web Surveys: The Checklist for Reporting Results of Internet E-Surveys (CHERRIES). J Med Internet Res (2004) 6(3):e34–e. doi: 10.2196/jmir.6.3.e34 PMC1550605 15471760

[16] Von Elm E Altman DG Egger M Pocock SJ Gøtzsche PC Vandenbroucke JP. The Strengthening the Reporting of Observational Studies in Epidemiology (STROBE) Statement: Guidelines for Reporting Observational Studies. J Clin Epidemiol (2008) 61(4):344–9. doi: 10.1016/j.jclinepi.2007.11.008 18313558

[17] Kroenke K Spitzer RL Williams JB. The PHQ-9: Validity of a Brief Depression Severity Measure. J Gen Intern Med (2001) 16(9):606–13. doi: 10.1046/j.1525-1497.2001.016009606.x PMC1495268 11556941

[18] Spitzer RL Kroenke K Williams JB Group PHQPCS Group PHQPCS. Validation and Utility of a Self-Report Version of PRIME-MD: The PHQ Primary Care Study. JAMA (1999) 282(18):1737–44. doi: 10.1001/jama.282.18.1737 10568646

[19] Spitzer RL Williams JB Kroenke K Hornyak R McMurray J Group PHQO-GS. Validity and Utility of the PRIME-MD Patient Health Questionnaire in Assessment of 3000 Obstetric-Gynecologic Patients: The PRIME-MD Patient Health Questionnaire Obstetrics-Gynecology Study. Am J Obstet Gynecol (2000) 183(3):759–69. doi: 10.1067/mob.2000.106580 10992206

[20] Spitzer RL Kroenke K Williams JB Löwe B. A Brief Measure for Assessing Generalized Anxiety Disorder: The GAD-7. Arch Intern Med (2006) 166(10):1092–7. doi: 10.1001/archinte.166.10.1092 16717171

[21] Hinz A Klein AM Brähler E Glaesmer H Luck T Riedel-Heller SG. Psychometric Evaluation of the Generalized Anxiety Disorder Screener GAD-7, Based on a Large German General Population Sample. J Affect Disord (2017) 210:338–44. doi: 10.1016/j.jad.2016.12.012 28088111

[22] Buysse DJ Reynolds CF III Monk TH Berman SR Kupfer DJ. The Pittsburgh Sleep Quality Index: A New Instrument for Psychiatric Practice and Research. Psychiat Res (1989) 28(2):193–213. doi: 10.1016/0165-1781(89)90047-4 2748771

[23] Hinz A Glaesmer H Brähler E Löffler M Engel C Enzenbach C. Sleep Quality in the General Population: Psychometric Properties of the Pittsburgh Sleep Quality Index, Derived From a German Community Sample of 9284 People. Sleep Med (2017) 30:57–63. doi: 10.1016/j.sleep.2016.03.008 28215264

[24] Ware JE Jr Kosinski M Keller SD. A 12-Item Short-Form Health Survey: Construction of Scales and Preliminary Tests of Reliability and Validity. Med Care (1996), 220–33. doi: 10.1097/00005650-199603000-00003 8628042

[25] Shim RS Baltrus P Ye J Rust G. Prevalence, Treatment, and Control of Depressive Symptoms in the United States: Results From the National Health and Nutrition Examination Survey (NHANES), 2005–2008. J Am Board Family Med (2011) 24(1):33–8. doi: 10.3122/jabfm.2011.01.100121 PMC3161724 21209342

[26] Pal L Bevilacqua K Zeitlian G Shu J Santoro N. Implications of Diminished Ovarian Reserve (DOR) Extend Well Beyond Reproductive Concerns. Menopause (2008) 15(6):1086–94. doi: 10.1097/gme.0b013e3181728467 18791485

[27] Chrousos GP Torpy DJ Gold PW. Interactions Between the Hypothalamic-Pituitary-Adrenal Axis and the Female Reproductive System: Clinical Implications. Ann Intern Med (1998) 129(3):229–40. doi: 10.7326/0003-4819-129-3-199808010-00012 9696732

[28] Vermeulen A. Environment, Human Reproduction, Menopause, and Andropause. Environ Health Perspect (1993) 101(suppl 2):91–100. doi: 10.1289/ehp.93101s291 PMC1519927 8243411

[29] Mayerhofer A Dissen GA Costa ME Ojeda SR. A Role for Neurotransmitters in Early Follicular Development: Induction of Functional Follicle-Stimulating Hormone Receptors in Newly Formed Follicles of the Rat Ovary. Endocrinology (1997) 138(8):3320–9. doi: 10.1210/endo.138.8.5335 9231784

[30] Marcus MD Loucks TL Berga SL. Psychological Correlates of Functional Hypothalamic Amenorrhea. Fertil Steril (2001) 76(2):310–6. doi: 10.1016/S0015-0282(01)01921-5 11476778

[31] Williams NI Berga SL Cameron JL. Synergism Between Psychosocial and Metabolic Stressors: Impact on Reproductive Function in Cynomolgus Monkeys. Am J Physiol-Endocrinol Metab (2007) 293(1):E270–E6. doi: 10.1152/ajpendo.00108.2007 17405827

[32] De Pergola G Tartagni M d’Angelo F Centoducati C Guida P Giorgio R. Abdominal Fat Accumulation, and Not Insulin Resistance, is Associated to Oligomenorrhea in Non-Hyperandrogenic Overweight/Obese Women. J Endocrinol Invest (2009) 32(2):98–101. doi: 10.1007/BF03345694 19411803

[33] Lim S Norman RJ Davies M Moran L. The Effect of Obesity on Polycystic Ovary Syndrome: A Systematic Review and Meta-Analysis. Obes Rev (2013) 14(2):95–109. doi: 10.1111/j.1467-789X.2012.01053.x 23114091

[34] Kahn M Sheppes G Sadeh A. Sleep and Emotions: Bidirectional Links and Underlying Mechanisms. Int J Psychophysiol (2013) 89(2):218–28. doi: 10.1016/j.ijpsycho.2013.05.010 23711996

[35] Tranoulis A Georgiou D Soldatou A Triantafyllidi V Loutradis D Michala L. Poor Sleep and High Anxiety Levels in Women With Functional Hypothalamic Amenorrhoea: A Wake-Up Call for Physicians? Eur J Obstet Gynecol Reprod Biol X (2019) 3:100035. doi: 10.1016/j.eurox.2019.100035 31403123 PMC6687383

[36] Roy D Belsham DD. Melatonin Receptor Activation Regulates GnRH Gene Expression and Secretion in GT1–7 GnRH Neurons: Signal Transduction Mechanisms. J Biol Chem (2002) 277(1):251–8. doi: 10.1074/jbc.M108890200 11684691

[37] Laughlin GA Loucks AB Yen S. Marked Augmentation of Nocturnal Melatonin Secretion in Amenorrheic Athletes, But Not in Cycling Athletes: Unaltered by Opioidergic or Dopaminergic Blockade. J Clin Endocrinol Metab (1991) 73(6):1321–6. doi: 10.1210/jcem-73-6-1321 1955514

[38] Gordon CM Ackerman KE Berga SL Kaplan JR Mastorakos G Misra M. Functional Hypothalamic Amenorrhea: An Endocrine Society Clinical Practice Guideline. J Clin Endocrinol Metab (2017) 102(5):1413–39. doi: 10.1210/jc.2017-00131 28368518

[39] Hartz A Barboriak PN Wong A Katayama KP Rimm AA. The Association of Obesity With Infertility and Related Menstural Abnormalities in Women. Int J Obes (1979) 3(1):57–73. 528119

[40] Yonkers KA O’Brien PMS Eriksson E. Premenstrual Syndrome. Lancet (2008) 371(9619):1200–10. doi: 10.1016/S0140-6736(08)60527-9 PMC3118460 18395582

[41] Landén M Eriksson E. How Does Premenstrual Dysphoric Disorder Relate to Depression and Anxiety Disorders? Depress Anxiety (2003) 17(3):122–9. doi: 10.1002/da.10089 12768646

[42] Yonkers KA. Anxiety Symptoms and Anxiety Disorders: How are They Related to Premenstrual Disorders? J Clin Psychiatry (1997) 58(3):62–9. 9133494

[43] Goldstein I Kim NN Clayton AH DeRogatis LR Giraldi A Parish SJ. Hypoactive Sexual Desire Disorder: International Society for the Study of Women’s Sexual Health (ISSWSH) Expert Consensus Panel Review. Mayo Clin Proc (2017) 92(1):114–28. doi: 10.1016/j.mayocp.2016.09.018 27916394

[44] Leiblum SR Koochaki PE Rodenberg CA Barton IP Rosen RC. Hypoactive Sexual Desire Disorder in Postmenopausal Women: US Results From the Women’s International Study of Health and Sexuality (WISHeS). Menopause (2006) 13(1):46–56. doi: 10.1097/01.gme.0000172596.76272.06 16607098

[45] Biddle AKMPHP West SLP D’Aloisio AAMSP Wheeler SBMPH Borisov NNP Thorp JMD. Hypoactive Sexual Desire Disorder in Postmenopausal Women: Quality of Life and Health Burden. Value Health (2009) 12(5):763–72. doi: 10.1111/j.1524-4733.2008.00483.x 19192259

[46] Schiavi MC Spina V Zullo MA Colagiovanni V Luffarelli P Rago R. Love in the Time of COVID-19: Sexual Function and Quality of Life Analysis During the Social Distancing Measures in a Group of Italian Reproductive-Age Women. J Sex Med (2020) 17(8):1407–13. doi: 10.1016/j.jsxm.2020.06.006 PMC7342024 32653391

[47] Caruso S Rapisarda AMC Minona P. Sexual Activity and Contraceptive Use During Social Distancing and Self-Isolation in the COVID-19 Pandemic. Eur J Contracept Reprod Health Care (2020) 25(6):445–8. doi: 10.1080/13625187.2020.1830965 33044107

[48] Kolstad HA Bonde JP Hjøllund NH Jensen TK Henriksen TB Ernst E. Menstrual Cycle Pattern and Fertility: A Prospective Follow-Up Study of Pregnancy and Early Embryonal Loss in 295 Couples Who Were Planning Their First Pregnancy. Fertil Steril (1999) 71(3):490–6. doi: 10.1016/s0015-0282(98)00474-9 10065787

[49] Small CM Manatunga AK Klein M Feigelson HS Dominguez CE McChesney R. Menstrual Cycle Characteristics: Associations With Fertility and Spontaneous Abortion. Epidemiology (2006) 17(1):52–60. doi: 10.1097/01.ede.0000190540.95748.e6 16357595

[50] Nguyen BT Pang RD Nelson AL Pearson JT Benhar Noccioli E Reissner HR. Detecting Variations in Ovulation and Menstruation During the COVID-19 Pandemic, Using Real-World Mobile App Data. PloS One (2021) 16(10):e0258314. doi: 10.1371/journal.pone.0258314 34669726 PMC8528316

[51] Small CM Manatunga AK Marcus M. Validity of Self-Reported Menstrual Cycle Length. Ann Epidemiol (2007) 17(3):163–70. doi: 10.1016/j.annepidem.2006.05.005 16882471

[52] Nikolaou CK Hankey CR Lean MEJ. Accuracy of on-Line Self-Reported Weights and Heights by Young Adults. Eur J Public Health (2017) 27(5):898–903. doi: 10.1093/eurpub/ckx077 28633350

[53] Pan D Sze S Minhas JS Bangash MN Pareek N Divall P. The Impact of Ethnicity on Clinical Outcomes in COVID-19: A Systematic Review. EClinicalMedicine (2020) 23:100404. doi: 10.1016/j.eclinm.2020.100404 32632416 PMC7267805

[54] Fitzpatrick KM Harris C Drawve G. Living in the Midst of Fear: Depressive Symptomatology Among US Adults During the COVID-19 Pandemic. Depress Anxiety (2020) 37(10):957–64. doi: 10.1002/da.23080 PMC7405392 32667117

[55] Noble S McLennan D Noble M Plunkett E Gutacker N Silk M Wright G. Data From: The English indices of deprivation. Crown Copyright. (2019).

[56] Goodman M Adams N Corneil T Kreukels B Motmans J Coleman E. Size and Distribution of Transgender and Gender Nonconfroming Populations: A Narrative Review. Endocrinol Metab Clin (2019) 48(2):303–21. doi: 10.1016/j.ecl.2019.01.001 31027541

[57] Toufexis D Rivarola MA Lara H Viau V. Stress and the Reproductive Axis. J Neuroendocrinol (2014) 26(9):573–86. doi: 10.1111/jne.12179 PMC4166402 25040027

