# Supplementary material for: Female Reproductive Health Disturbance Experienced During the COVID-19 Pandemic Correlates With Mental Health Disturbance and Sleep Quality
Source: Front Endocrinol (Lausanne). 2022 Apr 1;13:838886. doi: 10.3389/fendo.2022.838886 (PMC9010734; doi:10.3389/fendo.2022.838886)
Supplement: Supplementary file 1 [file Table_1.docx]

**Supplementary Table 1**

**Stressors**

‘Have you had any of the following stressors over the course of the pandemic?’

n = 1,335

| Stressor | n/% |
| --- | --- |
| Work stress/change in employment status | 753/56% |
| Family illness/bereavement | 300/22% |
| Difficulties providing or arranging childcare | 300/22% |
| Change in living situation | 268/20% |
| Family/partner conflict | 265/20% |
| Change in financial situation | 257/19% |
| Difficulties with home schooling | 245/18% |
| Difficulty accessing healthcare | 206/15% |

**Positive aspects**

‘Have you experienced any of the following positive aspects in relation to the pandemic?’

n = 1,335

| Positive aspect | n/% |
| --- | --- |
| Positive impact on personal relationships | 678/51% |
| Reduced commuting time | 571/43% |
| Improvement in personal finances | 535/40% |
| Increased free time | 496/37% |
| Overall positive lifestyle change | 337/25% |
| Positive reformation of career | 170/13% |
| Increased sense of community | 164/12% |
